# Inequities in dementia risk and recommandations for inequity stratifiers collection in research

**DOI:** 10.1002/alz70858_106045

**Published:** 2025-12-26

**Authors:** Stefanie A Tremblay, Vasvi Dhir, Isabel McDonald, Juhi Tulsi, Robert‐Paul Juster, Laurence Kirmayer, Martin Guhn, Nancy Mayo, Maiya R. Geddes

**Affiliations:** ^1^ McGill University, Montreal, QC, Canada; ^2^ The Neuro, Montreal, QC, Canada; ^3^ Université de Montréal, Montreal, QC, Canada; ^4^ Univeristy of British Columbia, Vancouver, BC, Canada; ^5^ McGill University Research Centre for Studies in Aging, McGill University, Montreal, QC, Canada; ^6^ The Neuro, Faculty of Medicine, McGill University, Montreal, QC, Canada

## Abstract

Certain communities, including ethnic and cultural minorities, experience disproportionately higher rates of dementia and worse outcomes.^1,2^ Structural and social determinants of health (SSDH)—the conditions in which people are born, live, work, and age^3^—are likely key drivers of these disparities.^4^ The effects of SSDH accumulate over the lifecourse, influencing physical, mental, and brain health, as well as the ability to adopt healthy behaviors.^4^ Despite growing recognition of their role in dementia inequities, SSDH remain inconsistently integrated into research, and Canada lacks specific guidelines for their inclusion in dementia studies.

This project seeks to provide clear guidance for integrating SSDH into aging and dementia research in Canada. In collaboration with a network of stakeholders and knowledge users, we are developing a recommendation framework grounded in community and expert input. To ensure a comprehensive approach, we are 1) **Engaging community partners** (e.g., individuals with lived experience, advocates for marginalized communities) through mind mapping sessions to identify the factors they view as most critical to maintaining brain health in aging. 2) **Conducting a Delphi survey** that invites both field experts and community partners to prioritize key SSDH indicators. 3) **Reviewing current practices** for assessing SSDH in Canadian longitudinal studies on dementia and aging through a scoping review, the findings of which will further inform the recommendations.

The resulting guidance framework will be shared as an online toolkit to facilitate its adoption and promote harmonization across Canadian cohorts. To support implementation, workshops and webinars will be organized to help researchers integrate SSDH into all stages of their work, from data collection to analysis and interpretation. Adopting a harmonized SSDH battery across Canadian cohorts will enable the generation of large, pooled datasets, allowing for robust investigations of SSDH variables and their interactions.

Although this initiative is tailored to the Canadian context, we hope it can serve as a model for other countries and be adapted to address dementia‐related inequities worldwide.

References

1. Lee et al. *JAMA Network Open*. 2022;5.

2. Mukadam et al. *Int. J. Geriatr. Psychiatry*. 2011;26.

3. Gómez et al. *JPHMP*. 2021;27.

4. Adkins‐Jackson et al. *A&D*. 2023;19.

